# Irony detection in patients with borderline personality disorder: an experimental study examining schizotypal traits, response biases and empathy

**DOI:** 10.1186/s40479-022-00194-w

**Published:** 2022-10-04

**Authors:** Anne Katrin Felsenheimer, Carolin Kieckhäfer, Alexander Michael Rapp

**Affiliations:** 1 Department of Psychiatry and Psychotherapy, University of Tübingen, 72076 Tübingen, Germany; 2 Max Planck School of Cognition , Max Planck Institut for Human Cognitive and Brain Sciences , Leipzig, Germany; 3 LVR Hospital Düsseldorf, Heinrich-Heine-University Düsseldorf, Düsseldorf, Germany

**Keywords:** Schizotypal personality, Sarcasm, Negativity bias, Social cognition, Pragmatic language, Mentalization, Nonliteral

## Abstract

**Background:**

In verbal irony we often convey meanings that oppose the literal words. To look behind these words, we need to integrate perspectives of ourselves, others, and their beliefs about us. Although patients with borderline personality disorder (BPD) experience problems in social cognition and schizotypal symptoms, research on irony comprehension mainly focused on the schizophrenic spectrum. Accounting for possible negative biases in BPD, the current study examined the detection of praising and critical irony in a text messaging interface.

**Methods:**

The cross-sectional study included 30 patients and 30 matched controls, who completed measures of cognitive and affective empathy (Interpersonal Reactivity Index, IRI), schizotypal (Schizotypal Personality Questionnaire; SPQ), and borderline symptoms (Borderline Symptom List; BSL-23) and the irony detection task. The irony task contained critical and praising remarks embedded in text messages. Asking for literality (ironic vs. literal) and intention ratings (critical to praising) of the stimuli, it allowed to analyze the sensitivity of literality detection as well as implicit and explicit response biases in a signal detection framework.

**Results:**

Borderline symptoms explained lower sensitivity for the detection of literal and ironic statements across groups. Whereas HC showed a negativity bias when implicitly asked about the literalness of the statement, patients with BPD perceived praising utterances as less praising when explicitly asked about their perceived intention. Neither empathy nor schizotypy explained outcomes beyond borderline symptoms.

**Conclusions:**

This was the first study to show lower detection of verbal irony in patients with BPD. While patients were less biased when asked about the literality of a statement, they perceived praising remarks as less positive on explicit measurements. The results highlight the importance of congruent, transparent communication in promoting epistemic trust in individuals with BPD.

**Supplementary Information:**

The online version contains supplementary material available at 10.1186/s40479-022-00194-w.

## Introduction

The psychopathology of borderline personality disorder (BPD) manifests in social interaction. In line with this, research on BPD has focused increasingly on the inferences people draw from these interactions, so-called social cognition [1–3]. One of the numerous concepts within the domain of social cognition is mentalization [4, 5]. It comprises the implicit and explicit understanding of oneself and others [6] and is developed in early social interactions throughout childhood [6]. Mentalization-based theory proposes an errant development of mentalization contributes to etiology of BPD [6, 7]. In a supportive environment, the caregiver shows the child that they are seen as an intentional being by empathetically mirroring the child’s expressed state of mind (e.g., crying). This helps the child internalize a coherent representation of self and others [6]. Additionally, caregivers provide ostensive cues (e.g., turn-taking or appropriate eye contact) to show that they are communicating socially relevant information [8]. It fosters epistemic trust, which is the general assumption that the information we receive from others is accurate, reliable and personally relevant. Epistemic trust assures us that we are not being intentionally misinformed and allows us to integrate information in our knowledge about the world [8–11]. A history of maltreatment and neglect may facilitate mistrust around communication in patients with BPD, which can make it harder to believe others [10, 11, 12]. That is, individuals with BPD experience childhood adversity 13 times more than non-clinical individuals, especially emotional abuse and neglect [13]. With an abusive parent, integrating given information can be dangerous [12] and those with BPD may overinterpret hostile motives when there are none. This form of "hypermentalization" [14–22] preserves and prolongs interpersonal conflicts [11].

To master the complexity of communication, we not only have to trust the information given to us, but also distrust it from time to time. A prime example is verbal irony, in which the vocal pitch or incongruent context suggest that the speaker intends the opposite of the literal words [23]. Impairments in the comprehension of irony has been mostly demonstrated for autism [24] and in the context of concretism in schizophrenia [25–30]. Being equipped with higher mistrust in the first place, it is likely that patients with BPD, too, may have difficulties to decide which information to trust in irony. In line with this and BPD’s eponymous description of the ‘border’ between psychosis and neurosis [31], individuals with BPD share cognitive biases with schizophrenia [32], show schizotypal traits [33], and/or psychotic symptoms [34–38]. Such a transdiagnostic symptomatology challenges the differential diagnostic specificity of nonliteral language deficits. Notably, in personality disorders (PD), BPD and schizotypal personality disorder (SPD) are known to co-occur [33, 39]. Hence, with regard to the dimensional alternative model for personality disorders (AMPD), which has been increasingly applied since the DSM V, the question arises as to which PD pathology is responsible for the ironic misinterpretation in previous studies of schizotypy [28, 40].

In irony, there are two causes of misinterpretation: not being able to detect the intention of the speaker, and being able to, but opting for the literal meaning regardless. The first cause is closely related to mentalization. Irony requires recognizing an intention hidden behind literal words. For this reason, it has been studied mainly in research on social cognition [41–43] and is used as its direct measure in video-based tasks [14, 44]. One of these tasks, the Movie for the Assessment of Social Cognition (MASC; [44]) has been widely applied in BPD [18, 45–47]. The MASC does not specifically examine irony, but uses ironic remarks among other scenarios as a measure of social cognition. Németh et al. [1] showed that in these multimodal tasks, individuals with BPD’s social cognition impairments are most pronounced [1, 48, 49]. Their response formats offer different interpretations of social situations [44], so selecting the right one requires the subject to explicitly compare different mental states [1]. In these tasks, participants with BPD demonstrate reasoning about mental states, but tend to overinterpret social cues [14–22]. By contrast, they show no impairments in nonverbal paradigms such as the Reading the Mind in the Eyes Task [50] which only requires to identify an emotion based on pictures of the eye region [1]. The authors concluded that the mere detection of emotions seems to be preserved in BPD. Instead, difficulties arise when multiple perspectives need to be explicitly compared. Multiple perspectives, however, are an inevitable part of irony [41, 51, 52]. And understanding irony requires a flexible shift between them - shifts that seems to be harder for individuals with BPD [53–55].

Yet, even the full capacity to compare mental states does not necessarily guarantee that a statement will be perceived as ironic. Irony explicitly leaves the intention of the speaker ambiguous and along that room for interpretation. Individuals who tend to perceive others as dishonest may decide to ignore irony, irrespective of their ability to infer mental states. Addressing this distinction in schizophrenia, Parola et al. [27] analyzed both sensitivity (the detection of a communicative intention) and response bias (the tendency to favor a specific response) during indirect speech comprehension. Individuals with schizophrenia had equal difficulty detecting ironic, deceitful, and sincere phrasings, but tended to perceive ironic utterances more deceitful than healthy controls. Negative attribution styles are common in BPD as well [2]; many individuals with BPD tend to interpret others’ behavior as aggressive and hostile [56] and neutral faces as less trustworthy [57]. This places patients in a vicious cycle of reliving traumatic relationships [12, 58]. Therapists are often encouraged to use clear, unambiguous communication to avoid unintentionally reinforcing the threat perceived by their patients [59]. This is especially true as negative biases in BPD tend to develop specifically in the face of ambiguous stimuli [3, 49, 60–62].

Using irony as a prime example of ambiguous language allows both pragmatic inference and attributional bias to be examined within one linguistic phenomenon. Most studies on irony comprehension, however, focus solely on ironic criticism or sarcasm [44], thereby confounding irony with an a priori negative bias. Analyzing both praising and critical irony bypasses positive testing and allows interpretation errors to be analyzed without overtly asking for them. For example, Kieckhäfer et al. [63] examined how the detection of ironic and literal praising and critical relate to borderline and schizotypal traits in healthy adults. In their study, both traits were associated with lower detection accuracy, though each set of traits had differing error patterns. In line with Parola et al.’s findings in schizophrenia [27], individuals with higher schizotypy interpreted the stimuli more mocking: They indicated literal praise as ironic critique and ironic praise as literal critique. In contrast, individuals with high borderline traits only made errors identifying ironic remarks and this was regardless of the intention.

We applied Kieckhaefer et al.’s [63] paradigm, for the first time, on participants diagnosed with BPD. We compared the detection of literality (ironic vs. literal) and implicit response biases within a signal detection theory (SDT) framework, as well as explicit ratings of the perceived intention (critical to praising) with healthy controls (HC). In accordance with findings on healthy adults with borderline symptoms [63], we hypothesized that participants with BPD would have more difficulty differentiating ironic and literal utterances. We further assumed that negativity biases would emerge in a more pronounced BPD symptomatology. Thus, in contrast to Kieckhäfer et al.’s results [63], we expected participants with BPD to interpret ironic praise and literal criticism literally, and ironic criticism and literal praise ironically. In line with this, we predicted BPD participants would rate critical remarks as more critical and praising remarks as less praising. To clarify the specific contributions of borderline and schizotypal symptoms on irony and to consider a more dimensional assessment of PD, we examined the relationship between these characteristics and irony comprehension across groups. Last, we included possible mentalizing capacities related to irony comprehension in BPD via affective and cognitive empathy.

## Methods

30 participants with BPD were recruited from the University Hospital of Tuebingen, Department of Psychiatry and Psychotherapy, Germany. The ward was specialized on dialectical behavioral therapy (DBT, [64]). General exclusion criteria were acute or anamnestic substance abuse or dependence, bipolar disorder, psychotic disorders, severe episodes of major depression, and neurological diseases. Inclusion criteria involved normal or corrected-to-normal vision, age between 18–55, native German speakers, and a clinical diagnosis of BPD for the patient group. A trained clinician assessed the DSM-IV criteria according to Structured Clinical Interview for DSM IV II (SCID II) [65] and comorbidities according to SCID I [66]. Except for 7 individuals, patients exhibited comorbid diagnoses, which included depressive disorders (*n* = 13), post traumatic stress disorder (*n* = 11), substance use but abstinent for at least 2 months (*n* = 1) and attention deficit hyperactivity disorder (*n* = 1). However, none of them fulfilled the diagnostic criteria for other personality disorders according to the traditional DSM-IV model. The study protocol was approved by the ethics committee of the Medical Faculty of the University of Tuebingen and carried out according to the Declaration of Helsinki. All participants provided written informed consent and received monetary compensation.

A group of 30 healthy controls (HC), was matched for age, verbal intelligence according to the multiple-choice vocabulary test (MWT, [67]), gender, and educational level. Both groups filled out the short version of the Borderline Symptom List (BSL-23, [68]) and the German version of the Schizotypal Personality Questionnaire (SPQ, [69, 70]). For the evaluation of cognitive and affective empathy, the Interpersonal Reactivity Index (IRI, [71]) was used as a German short version [72]. The IRI is a self-report instrument comprising two cognitive subscales (perspective taking, fantasy) and two affective subscales (empathic concern, personal distress).

After consenting to participate, demographics were assessed in paper-pencil format. Then, participants completed the irony paradigm and self-report instruments on a computer in a quiet, distraction-free room. The stimuli were the same as in Kieckhäfer et al. [63]; test construction and development are explained in detail there. Each trial consisted of a videotaped context story introducing a character in a café and subsequent message exchanges. According to the narrative, participants saw text messages containing a context sentence and a reaction to that message by the protagonist of the video (see Additional file 1). The message was either ironic praise (IP), ironic criticism (IC), literal praise (LP), or literal criticism (LC). In ironic stimuli, the intended meaning opposed the literal meaning. For example, IP had a praising intention by way of critique (“I went running today” “You are so lazy.”). Videos varied in the degree of proximal perspective, and were either addressed directly by the protagonist (2nd person) or observed by the protagonist talking to a neutral other (3rd person). The protagonist’s answers were to be scored on their literality (ironic vs. literal) in a binary response format, and their perceived intention (criticism vs. praise) on a five-point Likert scale (see Additional file 2). Each trial comprised five items per condition (20 items total). Participants completed two test versions, with one perspective each. Summation of correct identified items lead to a total maximum score of 10 correct responses per condition (IP, IC, LP, LC) for both test versions.

We applied SDT to quantify sensitivity (d’) and response biases (*β*). As in SDT designs, the irony task required a binary label of literality (literal vs. ironic), which could be compared to the presence or absence of a signal (irony present vs. irony absent), resulting in four logical outcomes (Table 1): hit (choosing ironic in an ironic stimulus), false alarm (choosing ironic in a literal stimulus), miss (choosing literal in an ironic stimulus), and correct rejection (choosing literal in a literal stimulus). Each category was assigned a likelihood ratio. For instance, the hit rate represents the proportion of ironic stimuli to which the participant responded “ironic”, and false alarm rate denotes the proportion of literal trials to which the participant responded “ironic”. Unlike the mere number of correct responses, SDT’s measure of sensitivity reflects the probability of identifying the intention of the stimulus while avoiding false alarms, and corresponds to the Z-value of the hit rate minus the false-alarm rate.

**Table 1:** Signal detection theory matrix with possible outcomes for each contrastive pair of stimuli

Stimulus pair	response	
	ironic	literal
IC vs. LP		
IC (irony present)	hit	miss
LP (irony absent)	false alarm	correct rejection
IP vs. LC		
IP (irony present)	hit	miss
LC (irony absent)	false alarm	correct rejection

*IC* ironic criticism, *IP* ironic praise, *LC* literal criticism, *LP* literal praise

SDT further accounts for the response bias *β*: a systematic criterion when a signal is considered as present. It can capture the tendency of an individual to interpret statements either as ironic or literal. An individual who tends to interpret statements as “ironic” shows high hits for ironic (IC and IP), but high false alarms in literal stimuli (LC and LP). An unbiased observer’s *β* is close to 1. With a tendency to respond “ironic” (liberal criterion), *β* approaches 0. With the tendency to choose “literal” (conservative criterion), *β* exceeds 1. d’ and *β* were computed with the R package psycho. The binary answer format (ironic vs. literal) and definition of irony as the opposite of the literal meaning resulted in two corresponding conditions (IP vs. LC; IC vs. LP). Specifically, in an IC stimulus (“I am too late.” “You are so reliable.”), the detection of the correct literality (i.e., “ironic”) requires detecting the critical intention, despite the literal praise. The same holds true for IP and LC for a praising intention. For each participant, we calculated the hits, false alarms, misses, and correct rejections for both matching pairs.

Then, we applied linear mixed effect models in R with the lme4 package using d’, *β,* and ratings of perceived intention as respective outcome; group (HC vs. BPD) and intention (praise vs. criticism) as sum-coded fixed effects; age and verbal intelligence as continuous covariates; gender as a categorical covariate; and random effects by participant. Post-hoc tests with adjusted p-values were carried out with Tukey’s test. Based on the stimulus design, misclassifying literality causes perception of the opposite intention (e.g., ironic praise as literal criticism). Thus, perceived intention was estimated by the mean rating of items correctly identified as ironic or literal.

For each model, the impact of borderline symptoms, schizotypal symptoms, and empathy scales was analyzed. Model fits were estimated hierarchically, starting out with the null model, then adding borderline and schizotypal symptoms, and finally IRI subscales, as fixed effects. Models were compared via Likelihood-ratio tests using the anova function.

Similar to previous results [63], the perspective of the speaker had no effect on detection performance in a preceding repeated measure Analysis of Variance (rmANOVA, see supplementary Table 1). Thus, the conditions were not included in analysis.

## Results

Groups did not differ significantly in age (*t* (58) = − .812, *p* = .420), gender (*Z* = .417, *p* = .519), educational level (*Z* = −1.736, *p* = .083), or verbal intelligence (*t* (58) = −1.062, *p* = .293). Patients with BPD had significantly more borderline symptoms (*t* (38.46) = −8.971, *p* < .001) and personal distress (*t* (58) = −6.215, *p* < .001), as well as lower perspective taking (*t* (58) = 2.871, *p* = .006) and more schizotypal symptoms (*t* (58) = −8.662, *p* < .001). A detailed sample description can be found in Table 2.

**Table 2:** Means (M) and standard deviations (SD) of demographic and psychometric data

	BPD (*n* = 30)		HC (*n* = 30)		*p*
	*M*	*SD*	*M*	*SD*	
Demographics					
age (years)	29.27	9.03	27.20	10.03	.420^a^
gender (female/male)	25/5		23/7		.519^b^
education (median/IQR)	4.00	1.25	4.00	0.00	.083^c^
verbal intelligence	28.51	4.07	28.89	3.71	.293^a^
Questionnaires^a^					
BSL-23	2.28	1.02	0.39	0.40	< .001
IRI					
personal distress	15.87	3.01	10.60	3.53	< .001
empathetic concern	15.63	2.47	14.73	2.60	.174
perspective taking	13.30	2.74	15.27	2.56	.006
fantasy	13.80	3.94	14.47	3.14	.472
SPQ	34.7	13.5	15.5	10.6	< .001
Perceived intention					
IC	2.25	0.62	2.17	0.46	
IP	3.56	0.69	4.01	0.47	
LC	1.94	0.49	1.81	0.42	
LP	4.52	0.37	4.70	0.25	
Sensitivity (d‘)					
IC vs. LP	1.11	0.31	1.17	0.21	
IP vs. LC	0.89	0.31	1.07	0.22	
Response bias (*β*)					
IC vs. LP	1.01	0.09	0.95	0.09	
IP vs. LC	1.07	0.11	1.13	0.09	

*HC* healthy controls, *BPD* borderline personality disorder, *BSL-23* Borderline Symptom List, *IRI* interpersonal reactivity index, *SPQ* schizotypal personality questionnaire, *IC* ironic criticism, *IP* ironic praise, *LC* literal criticism, *LP* literal praise

^a^independent sample t-test

^b^Pearson-Chi-Quadrat

^c^Mann-Whitney-U-Test

^d^Welch-Test

**Fig. 1:**
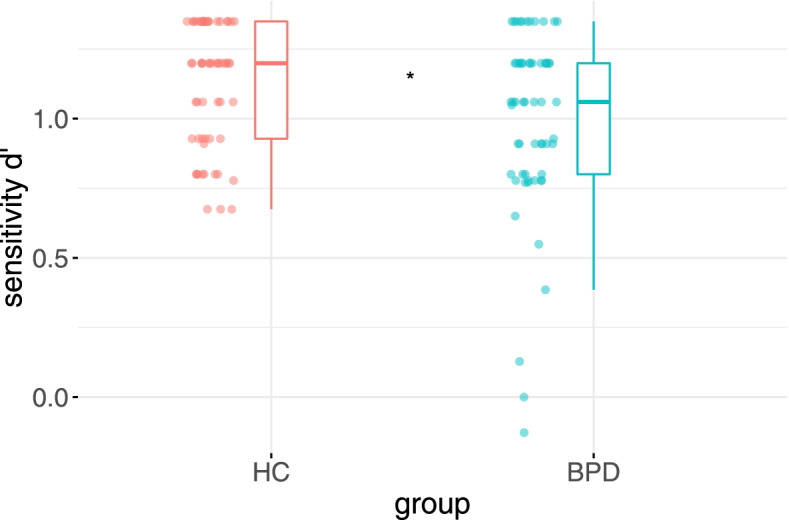
Sensitivity (dprime d') values for BPD and HC groups

Final models are depicted in Table 3. There was no effect of gender, age, or verbal IQ. Despite possible ceiling effects, patients with BPD (*M* = 1.00, *SD* = 0.32) showed significantly less sensitivity d' than HC (*M* = 1.12, *SD* = 0.22) in differentiating ironic and literal statements (*t* (58) = 2.184, *p* = .033), regardless of the intention (see Fig. 1). Above groups, sensitivity was higher for IC vs. LP (*M* = 1.14, *SD* = .27) than IP vs. LC (*M* = .98, *SD* = .28; *t* (58) = 4.22*, p* < .001).

**Table 3:** Analysis of deviance table (Type II Wald chi-square tests) for the linear mixed effect models with sum-coded contrasts and random intercepts by subject of sensitivity d' (left) and response bias *β* (right) including borderline symptoms

Fixed effects	sensitivity d’ ~ group*intention + bsl + age + gender + iq + (1|ID)				response bias *β* ~ group*intention + age + gender + iq + (1|ID)			
	*b*	*χ2*	*df*	*p*	*b*	*χ2*	*df*	*p*
group	0.00	0.01	1	.752	0.00	0.01	1	.923
intention	0.08	17.81	1	<.001***	−0.06	51.86	1	<.001***
group*intention	−0.03	2.31	1	.129	−0.03	10.90	1	<.001***
age	0.00	0.24	1	.623	0.11	0.73	1	.736
gender	0.05	0.62	1	.432	0.04	4.11	1	.042*
IQ	0.01	1.53	1	.216	−0.00	0.58	1	.446
BSL	−0.08	4.12	1	.042*				

*BSL* score on borderline symptom list 23

**Fig. 2:**
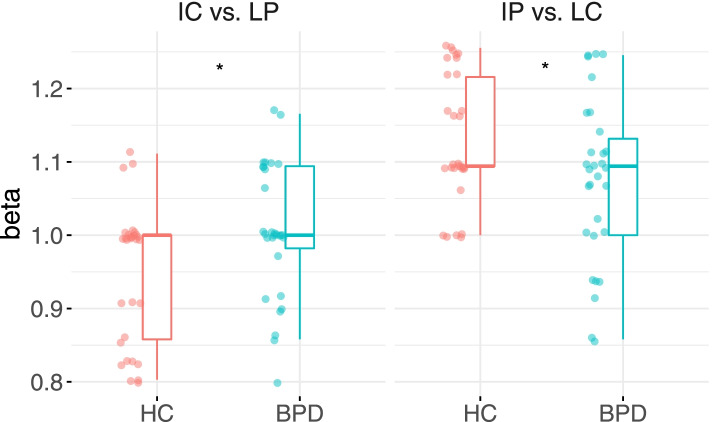
Response bias *β* for the comparisons of ironic criticism (IC) with literal praise (LP) and ironic praise (IP) with literal criticism (LC). An unbiased *β* corresponds to 1, a *β* approaching zero a tendency to choose ironic, a *β* increasing over one a tendency to choose literal as response

For response bias, there was a significant interaction of group on intention. However, post-hoc pairwise comparisons of group by level of intention indicated group differences in *β* for both IC vs. LP (*t* (116) = − 2.313, *p* = .023) and IP vs. LC (*t* (116) = 2.321, *p* = .022). On a descriptive level, *β* tended to be closer to 1 in BPD (see Table 1 and Fig. 2), indicating that BPD participants were almost unbiased. In contrast, HC showed a lower *β* in ironic criticism, corresponding with a tendency to interpret an answer as ironic in IC and LP and thus as mocking. The same negativity bias was evident in the other pair, with HC having a higher *β* in IP vs. LC and a tendency to choose literal. There was a significant effect of gender, with males having higher *β* than females (*t* (55) = − 2.027, *p* = .048).

Borderline symptoms significantly improved model fit for d’ (χ2 (1) = 5.497*, p* = .019), with a significant effect on d’ diminishing the effect of group. Neither SPQ (χ2 (1) = .187, *p* = .633), nor IRI subscales (χ2 (4) = 2.302, *p* = .680) improved model fit. For *β*, neither BSL (χ2 (1) = 0.011, *p* = .917), nor SPQ (χ2 (1) = 0.517, *p* = .472) or IRI scales (χ2 (1) = 0.064, *p* = .999) improved model fit.

In the last step we analyzed the ratings of perceived intention (Table 4). There was a significant interaction of group*intention, with clinical participants perceiving praising remarks as less praising (*t* (172) = 3.480, *p* < .001), but no difference in the perception of critical remarks (*t* (172) = −1.133, *p* = .259). Post-hoc comparisons for the interaction of intention*literal indicated all pairwise comparisons to be significant (all *p* < .0001), confirming previous findings that ironic remarks were perceived as less praising (IP: *M* = 2.21, *SD* = .63 vs. LP: *M* = 4.61, *SD* = .33) and less critical (IC: *M* = 2.21, *SD* = .54 vs LC: *M* = 1.87, *SD* = 46) in both groups. Again, neither BSL (χ2 (1) = 0.472, *p* = .491), SPQ (χ2 (1) = .740, *p* = .187), nor IRI scales (χ2 (1) = 5.017, *p* = .414) improved model fit.

**Table 4:** Analysis of deviance table (Type II Wald chi-square tests) for the linear mixed model of perceived intention with sum-coded contrasts and random intercepts by subject

fixed effects	perceived intention rating ~ group*intention*literality + (1|ID)			
	*estimate*	*X*^*2*^	*df*	*p*
between-subject				
group	0.053	2.72	1	.098
group*literality	0.042	1.74	1	0.187
group*intention	−0.103	10.77	1	< .001***
group*literality*intention	−0.027	0.75	1	.386
within-subject				
literality	−0.123	15.15	1	< .001***
intention	−1.076	1158.61	1	< .001***
intention*literality	0.290	84.45	1	< .001***

## Discussion

This was the first study to examine irony comprehension among individuals with BPD. Participants were presented with both ironic and literal text messages varying in praising and critical intention. Within a signal detection framework, we assessed response biases and the ability to discriminate literal from ironic remarks. Biases were distinguished on two levels: implicit tendencies measured in the choice of the literalness of the statement (ironic vs. literal) and explicit ratings of perceived intention (critical to praising).

Participants with BPD exhibited more difficulty differentiating literal from ironic remarks than HC. Yet, group differences did not vary with critical or praising content, showing that it was the literality of the stimulus, not the intention, affecting performance. For both groups, ironic praise was harder to detect than ironic criticism, replicating that ironic criticism is easier to process [73, 74], mostly because it is much more common [74–76]. The current results are commensurate with those in social cognition paradigms using sarcasm as a stimulus [15, 22, 46]. For the first time, these impairments have been confirmed with respect to verbal irony. Importantly, borderline symptoms explained reduced sensibility beyond categorical groups, confirming findings among healthy adults with borderline traits in a clinical sample [63] and corroborating dimensional approaches to personality disorders [77].

Other forms of nonliteral language, such as metaphors, have recently been shown to be preserved in BPD [78]. This is of particular importance, as metaphor comprehension is commonly impaired in schizophrenia [79] with whom BPD patients share symptoms [32, 33]. Contrary to other studies [28, 29, 40, 63, 80], schizotypal symptoms did not explain irony detection beyond borderline symptoms, although patients scored high on both. Our results support the idea that different forms of nonliteral language are subject to different cognitive processes [27, 81, 82]. And it implies that different expressions of psychopathologies may have their own causes of miscomprehension. For example, the cause of schizophrenic concretism has traditionally been understood as a difficulty with abstraction [83, 84]. In that sense, both metaphor and irony require an abstraction from the literal words, but irony further demands to integrate multiple mental states [8, 85, 86]. It is yet to explore whether specific sets of personality traits have their own processes hindering the comprehension of nonliteral language. To analyze this, there is a strong need to include assessments of abstraction (e.g., Wisconsin Card Sorting Test, [87]) and ecologically valid social cognition paradigms (e.g., The Awareness of Social Inference Test, [88]) as well as different forms of nonliteral language in future research.

Ironists do not intend to deceive but seek duplicitous understanding. As such, irony proves particularly challenging for mentalizing: It requires the listener to identify the other’s and own perspective, their relation and context. In our study, errors indicated that in some instances patients decided to stick to the literal meaning, even when an incongruence between context and target sentence suggested otherwise. Reduced mentalization may make it more difficult for individuals with BPD to decide which of these two meanings the speaker wants them to believe [10]. As a solution, they may adhere to one of them [16, 89] and choose a context-inappropriate interpretation. Indeed, shifts in the representation of the self and of others have long been deemed problematic in BPD [31]. They constitute the main personality psychopathology captured in Criterion A of the DSM-5 [90] and are largely represented by borderline symptoms [91]. Empirically, patients with BPD experience difficulties alternating between egocentric and altercentric perspectives with face-morphing tasks [54] and show overlapping self-other boundaries on a bodily and cognitive level [53, 54]. Accordingly, in our study, patient’s personal distress in response to others’ emotions was higher and cognitive perspective-taking lower than those of HC, replicating previous findings on self-reported empathy scales in BPD [92–94]. However, both were unrelated to outcomes in our study of irony. Future studies should include more complex social cognition paradigms that may be more commensurate with metacognitive processes than with self-ratings [48], and speech varying in self-other representation (e.g., deceit and faux pas). Instead of categorical groups, it will be essential to dimensionally assess impairments in self and interpersonal functioning (criterion A, [90]); e.g., via the Levels of Personality Functioning Scale (LPFS, [95]), as well as maladaptive personality variants (criterion B, [96, 97]).

Contrary to our expectations, HC (and not BPD) tended to interpret stimuli critically when deciding whether a remark was meant literally or ironically. Interpreting literal praise ironically HC ascribed negative intent to literal praise (“I have an A in my test” “You are clearly not smart”), while ironic criticism was seen as literal criticism. The same negativity bias was evident in ironic praise and literal criticism: HC tended to interpret these statements literally, considering ironic praise as literal critique and literal critique as such. What could be the reason for this? In our everyday conversations, ironic remarks usually express a critical attitude [74, 98, 99]. So, when asked to look for irony, a negative bias is a strategy that promises the most success. In terms of our cultural knowledge and experience, HCs decisions about whether or not to trust the literal remarks were therefore appropriate. Patients with BPD who grew up in an unreliable [6, 10, 11] communicative environment may not have developed such stable expectations about when interlocutors use what communicative intent [11]. And in a state of epistemic mistrust, a repeated experience such as “irony is typically negative” may not be internalized and generalized to other social contexts [11]. Instead, the trustworthiness of each new statement is assessed de novo. This can create uncertainty about what to expect and prevent efficient calibration of epistemic confidence, especially in ambiguous situations. Without stable priors, choosing whether to trust the negative (positive) context or the positive (negative) comment may thus be more random.

When it comes to explicit ratings of a critical or praising intent, the results are consistent with previous research on negativity biases. Both groups tended to rate ironic utterances as less praising and critical than literal ones, confirming the well-known perception of irony as “tinged” with the literal meaning [100, 101]. In contrast to implicit biases, patients with BPD perceived praising remarks as less praising than HC. So far, only a limited number of studies have investigated the effect of positive social stimuli in BPD [49]. Our findings are in line with BPD participants’ fear of positive appraisal [102], negative ratings of appreciating video-clips [103] or self-referential information [104] and approach-avoidance behavior [105, 106]. Muting the positive experience of praise has major implications, since positive feedback is a crucial part of the therapeutic process [107] and of positive interactions with others. Yet, contrary to other studies [103] and patients with BPD’s heightened rejection sensitivity [108] clinical participants did not differ to HC in perception of critical remarks.

The current study differs on multiple dimensions to tasks that often report a negativity bias [49, 109, 110]. First, it only included verbal stimuli. Negativity biases in BPD have mostly been found in non-verbal tasks such as facial emotion recognition [3, 49, 111, 112], especially in combination with other modalities [113, 114]. Second, we only assessed criticism and praise. Most biases in BPD regard anger and disgust [3, 111] or neutral stimuli [3, 61, 115]. Criticism only expresses dissatisfaction and less intense than anger. Further, irony is impossible to be neutral, as its principal function is to tacitly convey an opinion of the ironized content [52]. Third, implicit biases were assessed by asking participants to indicate the literalness of the stimulus (ironic vs. literal). In addition to explicit ratings of perceived intention, this allowed for a covert assessment of affective biases. In most emotion recognition paradigms, participants are asked directly about the emotion being displayed, which makes emotion as such the focus of attention and activates associated expectations about other people’s affective states. It may be that negativity biases are more pronounced when explicitly asked about the valence rather than the literalness of a statement.

We are aware of several limitations. First, they concern the generalizability of our sample. The patient sample had a high verbal IQ and educational background, which may be less prevalent in BPD among the general population [116, 117]. Patients were recruited in a specialized ward for DBT [118], which trains the differentiation between self and other and emotion regulation. On the one hand, this may have even minimized the group differences in irony detection. On the other hand, active practice of emotion regulation skills may have contributed to the unbiased response pattern in BPD compared to HC. This is further supported by the fact that borderline symptoms showed no association with response bias across groups. Despite inclusion and exclusion criteria, there was a high comorbidity of traditional Axis I disorders, resulting in heterogeneous psychopathology in the sample. Second, other limitations concern the applied paradigm. The difficulty of the stimuli seemed to be limited, which is why ceiling effect may have attenuated effects. Further, our stimuli did not contain nonverbal language such as prosody, facial expression or body posture. In line with this, the concept of irony transcends verbal irony, such as situational irony, hyperbole or understatement [119]. However, the study focused on the messenger interface which is a major part of current communication. Third, we did not account for experienced abuse or neglect, which is associated with epistemic distrust [10]. Lastly, this study did not include clinical controls, leaving the question of clinical specificity to be explored.

## Conclusions

This was the first study to provide evidence for an impaired irony detection in patients with BPD. Borderline symptoms explained this effect, but neither schizotypal traits nor empathy scales were related to outcomes of irony. While the use of ambiguous language is claimed to be restricted in therapeutic contexts with BPD patients [7, 107], the current study shows that this claim cannot be generalized to all forms of nonliteral language. With a preserved metaphor [78], but impaired irony comprehension in BPD, it seems that it is not the ambiguity of being nonliteral, but the ambiguity of the intention that imposes an obstacle for BPD. Just as irony forms a sense of collusion for those who understand [120], a patient’s misunderstanding may leave them with feelings of exclusion. In BPD this may even lead to a rupture in the therapeutic relation. Patients with BPD only showed a negative bias when explicitly asked to rate perceived intention. To them, praising remarks were considered less praising. Therapists and research alike naturally focus on negative social perceptions in BPD, but our results highlight the importance of targeting the diminished beneficial effect of positive feedback as well. Both findings emphasize the relevance of a shared and open discussion of the possible inferences BPD patients may draw from social interactions. But they also have significance for therapists. Together, they emphasize the need for practitioners to make their implicit mental states explicit, as encouraged in MBT [11, 107] and in DBT by specifying the dialectic [64, 118]. Our findings place a strong emphasis on MBT’s claim to encourage practitioners to be especially transparent, self-revealing, and explicit about their thoughts to promote epistemic trust, open the epistemic channel to integrate culturally and personally relevant information, and model the capacity for intentional communication in the patient. But they also stress the vital role of the therapist’s communication between the lines. Explicit mentalization is not a fully abstract process, but inherently interwoven with implicit, bodily intersubjectivity [121, 122]. Aligning words with intention and both with posture, prosody, and facial expression can serve to provide a clear basis for exploration of self and others in language.

## Supplementary Information

**Additional file 1.** Example stimulus presentation of ironic criticism.

**Additional file 2.** Dichotomous (literality) and rating scale (perceived intention) for each stimulus.

**Additional file 3: Supplementary Table 1.** Results of the rmANOVA of the dependent variables irony detection accuracy and perceived intention based on literality, intention, and perspective of presented stimuli. There were no group differences and interactions regarding the perspective the participant adopted with regard to the written dialogues.

## Data Availability

The datasets and material in the current study are available from the corresponding author upon request.

## Abbreviations

AMPD: Alternative Model of Personality Disorders
rmANOVA: Repeated measurements analysis of variance
BPD: Borderline personality disorder
BSL-23: Borderline Symptom List – short version
DSM-IV: Diagnostic and Statistical Manual of Mental Disorders
HC: Healthy control participants
IC: Ironic criticism
IP: Ironic praise
IRI: Interpersonal reactivity index
LC: Literal praise
LP: Literal criticism
MASC: Movie for the Assessment of Social Cognition
MWT: German multiple-choice vocabulary test
PD: Personality disorder
SCID-II: Structured Clinical Interview for axis II personality disorders
SCID-I: Structured Clinical Interview for axis I
SDT: Signal detection theory
SPD: Schizotypal personality disorder
SPQ: Schizotypal personality questionnaire
