# Supplementary figures and images for: Irony detection in patients with borderline personality disorder: an experimental study examining schizotypal traits, response biases and empathy
Source: Borderline Personal Disord Emot Dysregul. 2022 Oct 4;9:24. doi: 10.1186/s40479-022-00194-w (PMC9531442; doi:10.1186/s40479-022-00194-w)

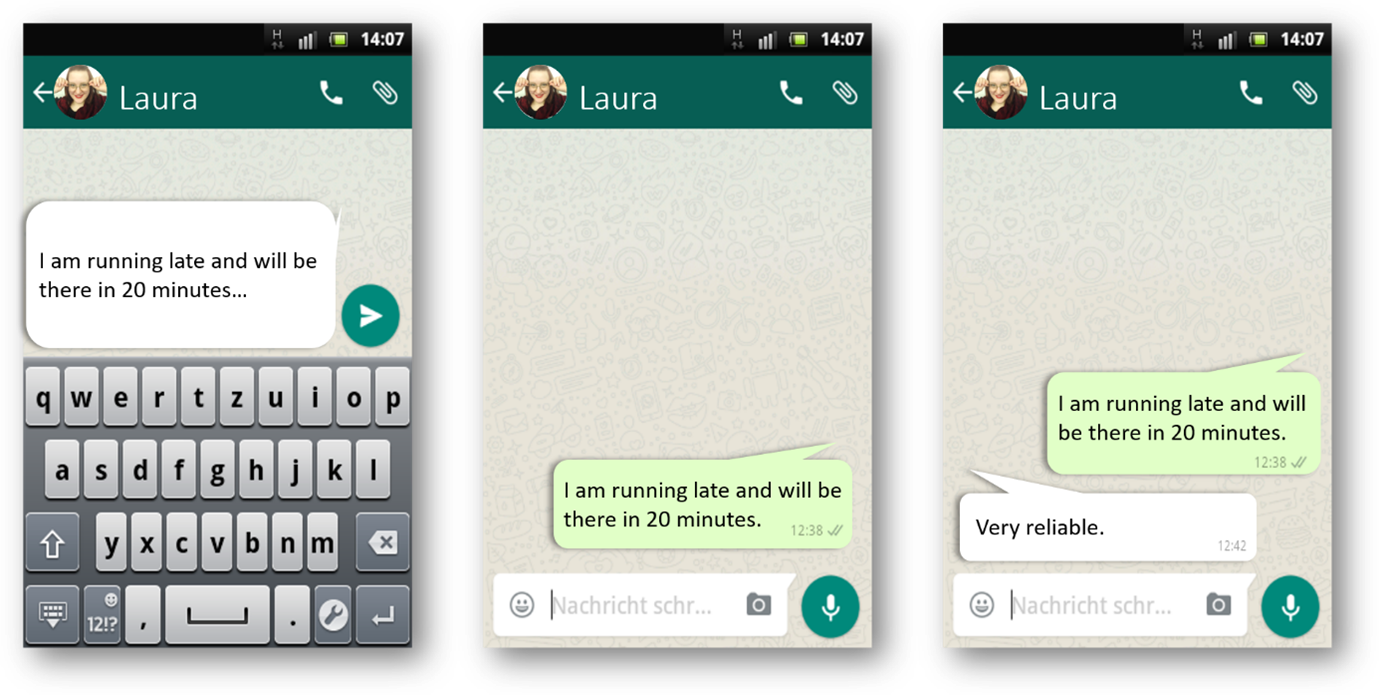

Supplement: Supplementary file 1 — Additional file 1. Example stimulus presentation of ironic criticism. [file 40479_2022_194_MOESM1_ESM.png]

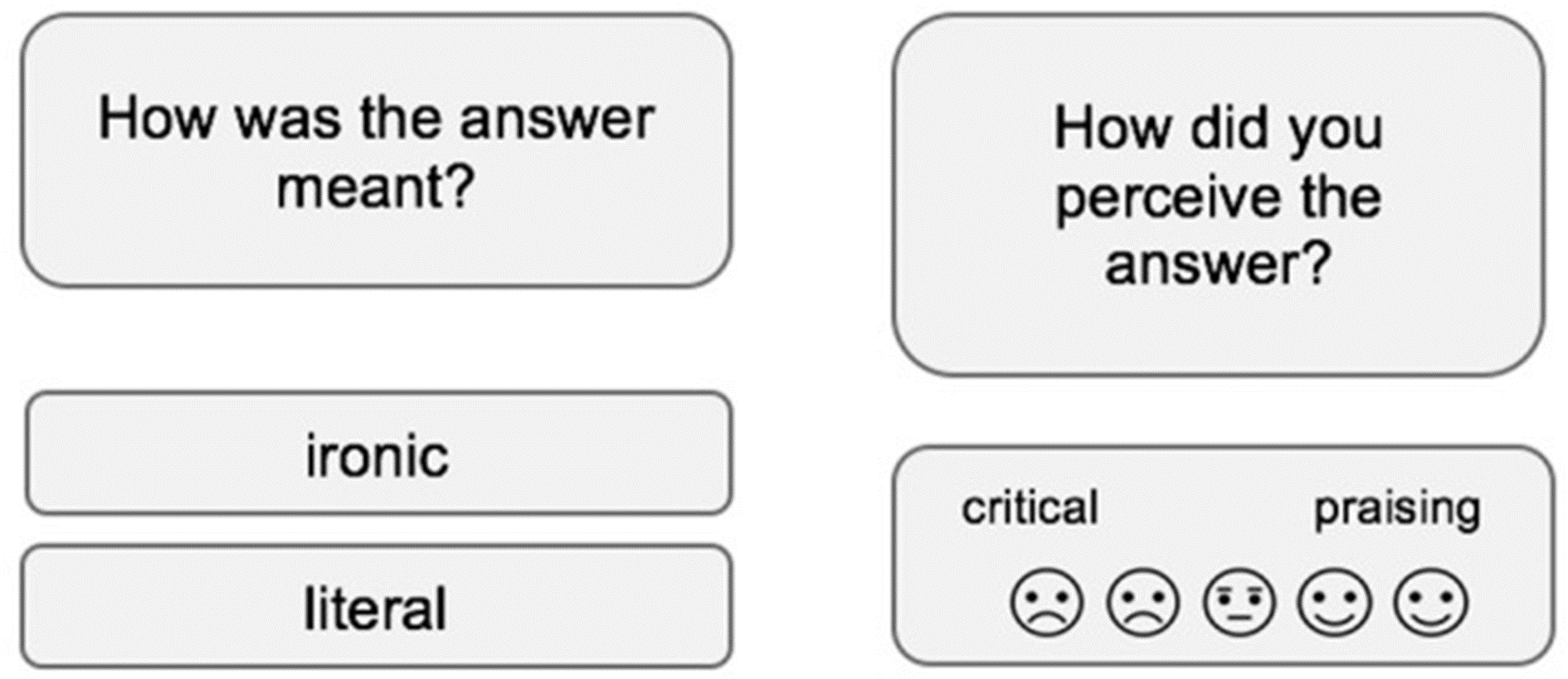

Supplement: Supplementary file 2 — Additional file 2. Dichotomous (literality) and rating scale (perceived intention) for each stimulus. [file 40479_2022_194_MOESM2_ESM.png]
