# Supplementary material for: Irony detection in patients with borderline personality disorder: an experimental study examining schizotypal traits, response biases and empathy
Source: Borderline Personal Disord Emot Dysregul. 2022 Oct 4;9:24. doi: 10.1186/s40479-022-00194-w (PMC9531442; doi:10.1186/s40479-022-00194-w)
Supplement: Supplementary file 3 — Additional file 3: Supplementary Table 1. Results of the rmANOVA of the dependent variables irony detection accuracy and perceived intention based on literality, intention, and perspective of presented stimuli. There were no group differences and interactions regarding the perspective the participant adopted with regard to the written dialogues. [file 40479_2022_194_MOESM3_ESM.docx]

**Supplementary Material**

Supplementary table 1. Results of the rmANOVA of the dependent variables irony detection accuracy and perceived intention based on literality, intention, and perspective of presented stimuli. There were no group differences and interactions regarding the perspective the participant adopted with regard to the written dialogues.

|  | accuracy | | | perceived intention | | |
| --- | --- | --- | --- | --- | --- | --- |
| factor | *F* | *p* | *partial η²* | *F* | *p* | *partial η²* |
| between-subject |  |  |  |  |  |  |
| group | 4.643 | .035* | .074 | .121 | .729 | .002 |
| group*perspective | 1.302 | .111 | .043 | .246 | .622 | .004 |
| group*literality | 4.602 | .023* | .086 | 4.447 | .039^*^ | .071 |
| group*intention | 1.957 | .167 | .033 | 14.442 | .000*** | .199 |
| group*literality*  intention | .666 | .418 | .011 | .452 | .504 | .008 |
| group*intention*  perspective | .006 | .936 | .000 | 3.125 | .078 | .053 |
| group*literality*  perspective | .036 | .850 | .001 | 2.240 | .140 | .037 |
| group*literality*  intention*perspective | .222 | .639 | .004 | .028 | .869 | .000 |
| within-subject |  |  |  |  |  |  |
| literality | 36.089 | .000*** | .384 | 24.086 | .000*** | .293 |
| intention | 16.603 | .000*** | .223 | 621.117 | .000*** | .915 |
| perspective | .004 | .949 | .000 | 4.22 | .043* | .069 |
| intention*literality | 20.995 | .000*** | .266 | 34.798 | .000*** | .375 |
| perspective*intention | .766 | .385 | .013 | 2.663 | .108 | .044 |
| perspective*literality | 1.765 | .189 | .030 | .093 | .761 | .002 |
| perspective*intention*  literality | .766 | .385 | .013 | 1.964 | .166 | .033 |

Additional file 1. Example stimulus presentation of ironic criticism.

Additional file 2. Dichotomous (literality) and rating scale (perceived intention) for each stimulus.
